# Efficacy of a training program to support the application of the *Guideline Evidence-based Health Information*: a randomized controlled trial and process evaluation

**DOI:** 10.1186/s13063-026-10057-2

**Published:** 2026-10-30

**Authors:** Julia Lühnen, Birte Berger-Höger, Burkhard Haastert, Jana Hinneburg, Jürgen Kasper, Anke Steckelberg

**Affiliations:** 1 Institute of Health, Midwifery and Nursing Science, Medical Faculty of Martin Luther University Halle-Wittenberg, University Medicine Halle, Magdeburger Straße 8, 06112 Halle (Saale), Germany; 2 Institute of Clinical Nursing Science, Charité – University Medical Center Berlin, corporate member of Freie Universität Berlin and Humboldt Universität zu Berlin, Charitéplatz 1, Berlin, 10117 Germany; 3 Institute for Public Health and Nursing Research, Faculty 11 Human and Health Sciences, University of Bremen, Grazer Str. 4, 28359 Bremen, Germany; 4 mediStatistica, Zur Waldesruh 25, 42329 Wuppertal, Germany; 5 Department of Nursing and Health Promotion, Faculty of Health Sciences, OsloMet: Metropolitan University, P.O. Box 4, St. Olavs plass, Oslo, 0130 Norway

**Keywords:** Health information, Guideline implementation, Training program, Evidence-based medicine, *Guideline Evidence-based Health Information*

## Abstract

**Background:**

The *Guideline Evidence-based Health Information* addresses health information providers and aims to improve the quality of health information.

This study aimed to evaluate the efficacy of a training program for health information providers to support the application of the guideline. We expected the intervention to improve the quality of health information compared with providing the guideline alone.

**Methods:**

In this superiority randomized controlled trial, providers responsible for producing and publishing health information were allocated 1:1 to the intervention (guideline and training program) and control (publicly available guideline) groups using computer-generated block randomization. The e-learning program (31 h, offered over 6–7 weeks) comprised modules on evidence-based medicine and the application of the guideline.

Each provider committed to producing a single piece of health information informing a health-related decision on the topic of their choice. The primary outcome was the quality of health information, operationalized as adherence to the guideline recommendations, as measured using the *Mapping Health Information Quality* (MAPPinfo) checklist. Means were compared between groups using a t-test. The assessment and analyses were blinded to the group allocation. An accompanying process evaluation was conducted.

**Results:**

We included 18 health information providers (nine in each group), with a total of 54 individual participants. Eight health information items were assessed in each group. The primary analysis estimated the mean MAPPinfo checklist values at 30.2% (95% CI, 24.6 to 35.9) for the intervention and at 22.7% (95% CI, 17.4 to 27.9) for the control group. The difference between groups was estimated at 7.6% (95% CI, − 0.2 to 15.3; *p* = 0.055). Individual and structural implementation barriers of the guideline recommendations were identified.

**Conclusions:**

We did not achieve the targeted sample size of 26 providers. A lack of resources was one of the main barriers to recruitment. Although our study did not provide conclusive evidence regarding the primary outcome, the participants rated the training program as relevant. Therefore, the training program will be made available to health information providers upon request.

**Trial registration:**

ISRCTN registry, ID: ISRCTN96941060. Registered on March 7, 2019.

Study protocol: https://doi.org/10.1186/s13063-020-04287-1 (Trials 2020).

**Supplementary Information:**

The online version contains supplementary material available at https://doi.org/10.1186/s13063-026-10057-2.

## Background

Evidence-based health information (EBHI) and patient decision aids are prerequisites for informed decision-making [1–3]. EBHI provides comprehensive, understandable, transparent, and unbiased information to support health decisions. It is essential that all options are presented, along with their potential benefits and harms. Patients’ right to EBHI is ethically required and, in many places, legally established [4–6]. However, the implementation of high-quality health information remains insufficient [7–9]. Various international and national initiatives have addressed this issue for several decades [10–12].

In Germany, the *Network for Evidence-based Medicine* released the second edition of the “*Good practice guidelines for health information”* in 2016 [13], which emphasized the need for standards for EBHI. Consequently, a project to develop an evidence-based guideline entitled *Guideline Evidence-based Health Information* was initiated. The guideline addresses health information providers and aims to improve the quality of health information [14]. The guideline was developed according to manuals and handbooks on guideline development [15–17], approaches to reform the guideline development processes to reduce bias [18–24], and the *Grading of Recommendations, Assessment, Development and Evaluation* (GRADE) approach [25]. The guideline development group comprised health information providers, health scientists, and patient representatives. The guideline and the methodological report were published in 2017 [14, 26].

Studies have repeatedly shown that medical guidelines may help improve practice [27–29]. In addition, training programs have been shown to support the implementation of guidelines [30]. As expected, the implementation of the *Guideline Evidence-based Health Information* was challenging. A qualitative study explored the competencies of health information providers in evidence-based medicine (EBM) and revealed relevant shortcomings [31]. To address these limitations, we developed a training program to promote the acquisition of EBM competencies and to introduce the application of the guideline [32].

### Objectives

This study aimed to evaluate the efficacy of a training program for health information providers to support the application of the *Guideline Evidence-based Health Information*. The central hypothesis of this study was that the intervention would improve the quality of health information compared with providing the guideline alone.

## Methods

This randomized controlled trial (RCT) followed the *CONsolidated Standards Of Reporting Trials* (CONSORT) 2025 Statement [33], the *UK Medical Research Council* (MRC) framework for the development and evaluation of complex interventions [34], and the *Template for Intervention Description and Replication* (TIDieR) checklist [35]. We involved an advisory board that included patients (*n* = 2) and health information providers (*n* = 4) in the development process of our study protocol. They advised particularly on the recruitment strategy. A detailed description of the methods is provided in the study protocol [36].

### Design

This was a superiority RCT that compared two groups of equal sizes. Additionally, we conducted a mixed-methods process evaluation to understand the mediating mechanisms and potential barriers to the implementation process.

### Setting and participants

This study was conducted between June 2019 and June 2022. The study’s target population was providers of publicly available health information in the German-speaking regions of Germany, Austria, and Switzerland. The providers were institutions or working groups comprising one or several individuals. The intervention was offered exclusively online, owing to the SARS-CoV-2 pandemic.

### Eligibility and recruitment

#### Inclusion and exclusion

We included health information providers who were responsible for the production and publication of health information and who published health information regularly and were currently active. The providers could participate if they were committed to producing health information within the next 10 months. Any members of the institution or working group closely involved in the development of the health information were also included and requested to attend the training.

We excluded members of the working groups on the *Guideline Evidence-based Health Information* [26] or the *good practice guidelines for health information* [13] or providers exclusively producing drug fact boxes or offering counseling services.

#### Recruitment

We conducted Internet searches using Google and MetaGer to identify health information providers. We applied different search terms to identify the online health information and webpages referring to this information. The provider data were extracted from the websites. We also included well-known providers. Subsequently, we assessed eligibility and sent out an invitation to participate in our study with a cover letter briefly informing the providers about the study and the *Guideline Evidence-based Health Information*. If no response was received, the providers were contacted by telephone and provided further information. If providers expressed interest in our study, we arranged a telephone or an online meeting to assess their eligibility.

If eligible, the providers were invited to a kickoff meeting that included further information about the study, a baseline assessment, and allocation to the intervention group (IG) or the control group (CG). Written informed consent and individual baseline data were obtained from participants prior to the kickoff meeting. Recruitment and randomization were performed consecutively.

#### Randomization and blinding

Participating provider groups were randomly assigned to either the IG or CG using a computer-generated list with randomly permuted blocks of length 2, 4, 6, or 8. The random allocation sequence was generated by a biometrician (BH) and safely transferred to an independent external person who prepared sealed, opaque envelopes. After the baseline assessment of the respective provider group was completed, the researchers opened the sealed opaque envelope to reveal the provider group’s allocation.

Owing to the nature of the intervention, allocation to the IG or CG was known to the providers and researchers conducting the training program. However, the assessment of the primary outcome and all analyses were blinded to group allocation.

### Interventions

#### Intervention group

The intervention comprised a training program aimed at facilitating the appropriate use of the *Guideline Evidence-based Health Information*.

The *Guideline Evidence-based Health Information* contains 21 evidence-based recommendations assigned to the following topics: presentation of frequencies; application of graphics, pictures, and drawings; narratives; value clarification tools; formats; and involvement of the target group. In addition, it addresses the general and ethical requirements regarding the development process, target group orientation, and content of EBHI. The guideline is publicly available (www.leitlinie-gesundheitsinformation.de).

##### Training program on the implementation of the Guideline Evidence-based Health Information

A training program, *From Guideline to Evidence-Based Health Information* (Guide2Info), was developed and pilot-tested in a blended-learning format. It comprises two modules (Module 1: EBM training, Module 2: Application of the *Guideline Evidence-based Health Information*). Further information on the underlying theories, development processes, and pilot testing has been published elsewhere [32].

Before the first training course, the onset of the SARS-CoV-2 pandemic made face-to-face training sessions impossible. Therefore, the training was adapted to a purely e-learning format, comprising 31 h offered over a period of 6–7 weeks. The training course was divided into self-study time (e.g., videos, texts, work assignments) and three to four synchronous online meetings via *Webex* (www.webex.com/de/index.html) (90–120 min each). We used the learning management system ILIAS, hosted by the *Halle School of Healthcare*, for the training course (www.ilias.de). The participants needed Internet access and had to register with a username and password at the *Halle School of Healthcare*. For the synchronous online meeting, a microphone and a camera were required. The content and module structure were retained during the adaptation. Methods and media that were mainly oriented toward the face-to-face classroom phase were extensively revised. For example, presentations were made available as screencasts, and content was now conveyed via texts or supplementary sources and tools. Work assignments were adapted so that virtual group work was possible. The reading and critical appraisal of the studies and the concrete application of guideline recommendations as core elements were fully retained. An overview of the objectives, methods, and contents is presented in Table 1. The training was provided by BBH and JLü, postdoctoral researchers in health and nursing science with expertise in EBM and EBHI and in teaching.

**Table 1:** Aims, methods, and content of the training program Guide2Info

Modules	Aims	Formats / Methods	Contents
**Module 1: EBM training**			
**Sub-module 1: introduction**	Participants gain an overview of the process of developing EBHI and reflect on their own approach. Participants begin to understand EBHI as a prerequisite for informed choices.	E-lectures	Introduction (e-learning platform, course of the training); presentation of the case study (focus on smoking cessation)
		Work assignments	1. Outline for the development of health information 2. Comparison of two pieces of health information
		Virtual presence	Introduction; clarification of confidentiality; debriefing online phase and work assignments 1 and 2; definition of EBHI, prospect
**Sub-module 2: treatment studies**	Participants understand the difference between association and causality and that RCT are suitable for showing causal relationships. Participants can describe the characteristics of RCT. Participants can critically appraise RCT and interpret the results.	E-lectures	Introduction to reliable information; RCT: design, methodology, risk of bias, interpretation of results (effect sizes, significance, confidence intervals)
		Media	Video on clinical studies
		Work assignments	3. Development of a study design for proof of efficacy 4. Data extraction from an RCT extract
		Exercises and discussion in the forum	Absolute vs. relative risk reduction; calculations using the four-field table
		Virtual presence	Debriefing work assignment 4, critical discussion of the study
**Sub-module 3: evidence-syntheses and guidelines**	Participants can critically appraise systematic reviews and meta-analyses and interpret the results. Participants describe the development process of guidelines and name their limitations.	E-lectures	Aim, definition and methodology of systematic reviews; structure and critical appraisal of medical guidelines
		Media	Podcasts on systematic reviews and publication bias
		Work assignments	5. Data extraction and critical appraisal, extract from a Cochrane Review 6. Survey: use of guidelines in the development of health information
		Virtual presence	Debriefing work assignments 5 and 6
**Sub-module 4: systematic literature search**	Participants can conduct systematic literature searches to identify relevant evidence for their questions.	E-lectures	Systematic literature searches; presentation of relevant databases
		Media	links to tutorials and guides on databases and literature searches
		Work assignments; peer feedback and feedback from lecturers	7. Survey: procedure for literature research 8. Creation of a search string for a given question 9. Research on your own question, peer feedback and feedback from lecturers
**Sub-module 5: diagnostic studies**	Participants can recognize the most important study designs for studies on diagnostic tests. Participants can calculate and interpret the values for test quality. Participants recognize the problem of overdiagnosis and overtreatment.	E-lectures	Quality criteria for diagnostic tests; studies on quality of diagnostic tests
		Media	Video on overdiagnosis
		Exercises	Survey: ‘Which test is absolutely reliable?’; test quality: calculations four-field table and layperson-friendly presentation
**Module 2: Application of the *Guideline Evidence-based Health Information***			
	Participants can develop EBHI and document the development process. Participants know strategies for piloting EBHI and can apply them. Participants understand EBHI as a prerequisite for informed choices.	E-lectures	Orientation in the *Guideline Evidence-based Health Information*; practical implementation of quality criteria; EBHI development process
		Work assignments; peer feedback	10. Development of excerpts of EBHI on selected topics (risk presentation based on original data), peer feedback 11. Selection of suitable methods of user testing
		Exercises; personal reflection and discussion in the forum	Critical evaluation of an EBHI; risk calculation and communication
		Virtual presence	Reflection on EBHI development processes; debriefing of work assignments 10 and 11; evaluation and implementation of EBHI; discussion of feasibility of the guideline criteria

In addition: provision of self-learning materials and further resources

Usability tests were conducted with a teacher experienced in e-learning and two students from health and nursing sciences. Data were collected through online interviews and written feedback. Optimization needs were identified, particularly regarding the structure and navigation between tasks and content on the learning platform. Adjustments were made, where technically possible.

#### Control group

Providers assigned to the CG received optimized standard care, which included access to the *Guideline Evidence-based Health Information*. Access to the training program Guide2Info was not provided until the CG had finished the study and data collection (primary and secondary outcomes) was completed.

### Baseline data

#### Baseline characteristics

We assessed the baseline characteristics of the health information providers at the institutional and individual levels. To survey the baseline characteristics at the institutional level, we conducted interviews at the kickoff meeting. We assessed the size of the organization, the number of health information publications, the main target group(s) and topic(s) of health information, the development processes, the funding of health information, and the management of conflicts of interest. The baseline characteristics of the participants were surveyed using a standardized questionnaire. We assessed their sex, age, educational status, English language skills, qualifications for the development of health information, duration of experience in developing health information, and previous experiences and attitudes regarding EBM and EBHI. In addition, a declaration of conflicts of interest was requested.

#### Baseline variables

Quality of health information: We requested that each provider submit the most recent piece of health information that had to meet the criteria for materials providing information on health-related decisions. The quality of the information provided was rated using the *Mapping Health Information Quality* (MAPPinfo) checklist [37]. Providers who produced different materials in a short time were allowed to select one piece of health information for the baseline assessment.

Critical health literacy: Critical health literacy was assessed using the *Critical Health Competence* (CHC) test [38]. The CHC test comprises four scenarios that allow testing of different scenarios at different time points. The test was conducted at baseline (scenario 1) and as part of the process evaluation after the training program in the IG and CG (scenario 3).

### Outcomes and data collection

#### Primary outcome

The primary outcome was the quality of health information, measured using the MAPPinfo checklist [37, 39]. The MAPPinfo checklist operationalized the guideline’s recommendations [37]. The instrument comprises 21–23 test items (depending on the nature of the addressed decision) in four categories:

- DEFINITIONS (target group [D1] and aim of the health information [D2])
- TRANSPARENCY (disclosure of authors [T1], financing [T2], conflicts of interest [T3], level of actuality [T4], sources of information [T5], and strategies for information gathering [T6])
- CONTENT (explanations of the medical problem [C1], available options [C2], uncertainty [C3], information about prevalence [C4], benefit [C5], harms [C6], and test quality if relevant [C7]).
- PRESENTATION (comprising prevalence [P1], benefits [P2], harms [P3], test quality if relevant [P4], language [P5], patient stories [P6], graphics if relevant [P7], and gain–loss framing [P8])

The items were coded as fulfilled (100%) or not fulfilled (0%), some also as partly fulfilled (50%). Scores for information quality were generated (range, 0–100%) at item level and as an average over all items. According to the MAPPinfo checklist [39], different aims within one piece of health information were assessed separately, and the highest rating was selected for further analyses. Information coding was performed by two independent and blinded raters (SZ and JLau) who had experience with the tool. Any disagreements were resolved through discussion. The raters received the health information under a pseudonym after data collection was completed.

To assess the primary outcome, the providers had to submit one piece of health information that fulfilled the following criteria: (1) inform a decision in health care (e.g., therapy, diagnostic procedure, screening, prevention, or rehabilitation), (2) address patients or medical laypersons, and (3) discuss at least two options regarding a specified health problem. Fact boxes without additional text were considered inappropriate. The providers were free to choose the topic and target group of the health information that had to be developed during the study. They were allowed to use external services (e.g., counseling by experts, graphic design) for the development process.

#### Secondary outcomes

1. Individual criteria of the MAPPinfo checklist [37] were used as secondary outcomes:

- CONTENT 5/PRESENTATION 2 (C5/P2): Information on possible benefits is provided in an appropriate manner.
- CONTENT 6/PRESENTATION 3 (C6/P3): Information on possible harm is provided in an appropriate manner.
- CONTENT 7/PRESENTATION 4 (C7/P4): In the case of diagnostic problems, information on the reliability and safety of the test is provided in an appropriate manner.
- PRESENTATION 5 (P5): Health information uses neutral wording and language throughout.

2) Involvement of the target groups in the development process: Involving the target group in the development process was part of the MAPPinfo self-declaration, which was named MAPPinfo-plus in a validation study [37]. It is not included in the MAPPinfo checklist itself, as the checklist is designed as a screening instrument. Therefore, the question regarding the involvement of the target group was integrated into the interviews with the providers at baseline (CG) and in the process evaluation (IG).

All secondary outcomes were binary.

### Sample size

The sample size was calculated for a t-test comparing the IG and CG regarding the quality of health information, as measured using MAPPinfo assuming a normal distribution and a standard deviation (SD) (σ) of 10% in both groups. Pretest data from MAPPinfo showed very low ratings for available online health information. The quality of this information can be improved with minimal effort. Based on these assumptions and a sample size of 26 provider groups (13 in each study group), a quality improvement of 1.5 × σ corresponding to a very strong Cohen’s effect size could be detected with 90% power using a two-sided significance level of 5% after excluding at most 10% of participants as dropouts. Power was calculated using the statistical software package SAS version 9.4 (PROC POWER).

### Procedure

After enrollment and allocation, a timely and convenient appointment for the training program Guide2Info was made for the IG. The training took place online and was scheduled for approximately 6–7 weeks, with dates for synchronous online meetings. The training period could be extended upon request. After the training was completed, the participants were allowed to start producing their health information. The providers in the CG started producing their health information immediately after randomization. During the time of production, we contacted the providers every 2 months to avoid overlooking problems that might have occurred. Additionally, the study team was available to answer any questions regarding the course of the study. Providers in both the IG and CG received support for the acquisition of research articles. No further support was provided for this study. The providers were requested to submit the developed health information within 10 months. This deadline could be extended by 2 months. However, if providers could justify further delays and present a reasonable timetable, the deadline could be extended beyond these 2 months. At the end of the study period, the unfinished materials had to be submitted.

After the submission of their health information, providers in the IG were interviewed to obtain further information on the development process and to explore barriers to and facilitators of the implementation of the guideline’s recommendations (cf. process evaluation). For the CG, we assumed that the methodological approach in the study context would not significantly differ from the usual approach, so that important information, such as sources of information and research strategies, was already collected in the interviews at baseline. Finally, the providers in the CG were offered the training program Guide2Info. All the providers received individual feedback on their health information.

### Data analyses

Provider characteristics (institutional and individual levels) were descriptively analyzed. The baseline characteristics were described at the individual level using means, SDs, and frequency tables. The IG and CG were described separately.

Critical health literacy: We assessed critical health literacy using the CHC test [36]. To analyze the CHC test, we calculated person parameters ± SD for participating individuals and mean values for IG and CG. Person parameters range from 0 to 1000. High person parameters reflect a higher level of critical health literacy.

The primary analyses were performed from an intention-to-treat perspective. The primary outcome was analyzed at the provider level, assuming an approximately normal distribution, by comparing the means of the IG and CG using an unpaired *t*-test. Because of the small sample size, the distribution of MAPPinfo scores was investigated graphically (not shown because of the low sample size). With respect to possible deviations from a normal distribution, a nonparametric exact Wilcoxon test was performed as a secondary sensitivity analysis. The frequencies of missing values were described, and multiple imputations were performed for the primary outcome. A monotone multiple imputation model (50 imputations) was fitted, including group, baseline MAPPinfo score, number of participants per institution, and mean baseline CHC at the institution as covariates. Predictive mean matching was used to ensure that only values within a range of existing MAPPinfo values were achieved. After multiple imputations, the mean values were estimated with 95% CIs for the IG and CG and for the differences between the groups. No specific protocol criteria were defined, and no protocol violations were observed. A secondary complete-case analysis was performed. To adjust for initial values, an ANCOVA model was fitted with dependent variable MAPPinfo and independent variables group (IG versus CG) and baseline values of MAPPinfo. In addition, we displayed means at the item level for the IG and CG using the MAPPinfo Evaluation Toolkit [40]. For binary secondary outcomes, the relative frequencies were described in both groups.

### Process evaluation

A convergent mixed-methods parallel approach was applied [41] according to the MRC framework for the process analyses of complex interventions [42].

We used structured documentation to assess data on recruitment and intervention fidelity (e.g., recruitment process, the number of institutions invited, responses and participants in each training session, location, time and duration of the training, completeness of modules and online tasks, and reasons for deviations, and unexpected difficulties). The feasibility and acceptance of the training program Guide2Info were assessed at the end of the training sessions using structured feedback. Critical health literacy was assessed using the CHC test [38] after the IG and CG completed the training program. We calculated person parameters ± SD for participating individuals at baseline and mean post-test values for the IG and CG. Descriptive statistics were used for the quantitative data.

To map the development process and methodological quality of the health information produced, providers of the IG were asked questions that had been taken from MAPPinfo-plus [37]. In the CG, it was assumed that the methodological procedure in the study context would not significantly differ from the usual procedure; therefore, important information, such as sources of information and research strategies, was already collected in the baseline interviews.

In addition, the resources and support used in the development process and any training attended during the study period were assessed in the interviews to identify influencing factors.

In the IG, we conducted semi-structured interviews with individual participants and with persons in charge to explore the barriers and facilitators of the implementation. In addition, we explored the factors relevant to the acceptance and usability of the training program. We were interested in the use of the training program in daily routines and the barriers experienced during the implementation of guideline recommendations. The interviews were audio-recorded and transcribed verbatim. The analyses comprised two methodological procedures.

I. Comparison of the development process with the process presented in the training

Learning objectives were defined for the training (Table 1). By achieving these learning objectives, the participants should be able to define a standard for the development of EBHI in accordance with the *Guideline Evidence-based Health Information*. The following developmental steps were reviewed: people involved in the development process, identification and quality assessment of existing information materials on the topic, exploration of the preferences of the target group, systematic identification of the evidence base, appropriate quality assessment of studies, systematic data extraction, piloting of the health information in the target group, and reflection on the procedure.

Two authors (JLü and AS) searched the transcripts for suggestions regarding the development process. These were assigned to the individual process steps. The results were summarized for all transcripts. Information identified in the method reports was included, particularly when referred to during the interviews. The submission of a method report was voluntary, and therefore, available only to some groups.

II. Content analysis

The content analyses of the transcripts followed the modified procedure described by Mayring [43]. After an initial review of the material, the categories and subcategories were established and agreed upon (JLü and AS). Next, all transcripts were coded using these categories. New subcategories were included. The final category system was agreed upon by all raters.

### Deviations from the study protocol

There were some deviations from the study protocol that were attributed to the SARS-CoV-2 pandemic and the recruitment challenges. With the exception of the changes to the training, the deviations affected both study groups equally. From the end of March 2020 on, the kickoffs, including information on the project, informed consent, a survey of baseline data, and allocation to the IG or CG, had to be switched from on-site at the provider groups to online meetings. The switch also affected the training. The training was converted into an e-learning format. We used *Webex* and *Lime Survey* for the meetings and a survey of data.

The workshop for persons in charge of providing health information (module 0) did not take place. It was intended to discuss and reflect on the benefits of using the EBHI criteria. However, the persons concerned either participated directly in the training or were not involved in the study inclusion at all.

To address the recruitment challenges, we also included providers without a history of published health information. They demonstrated a high level of motivation and provided concrete plans for developing health information. We also had to adapt the envisaged timeframe to develop the health information. We planned to prepare the information for a maximum of 10 months, or 12 months after the consultation. This deadline was waived to account for the generally higher workload caused by the SARS-CoV-2 pandemic on the one hand and to obtain the most complete information possible for the small sample on the other hand. The most recent information was requested for the preparation of the report.

## Results

**Fig. 1:**
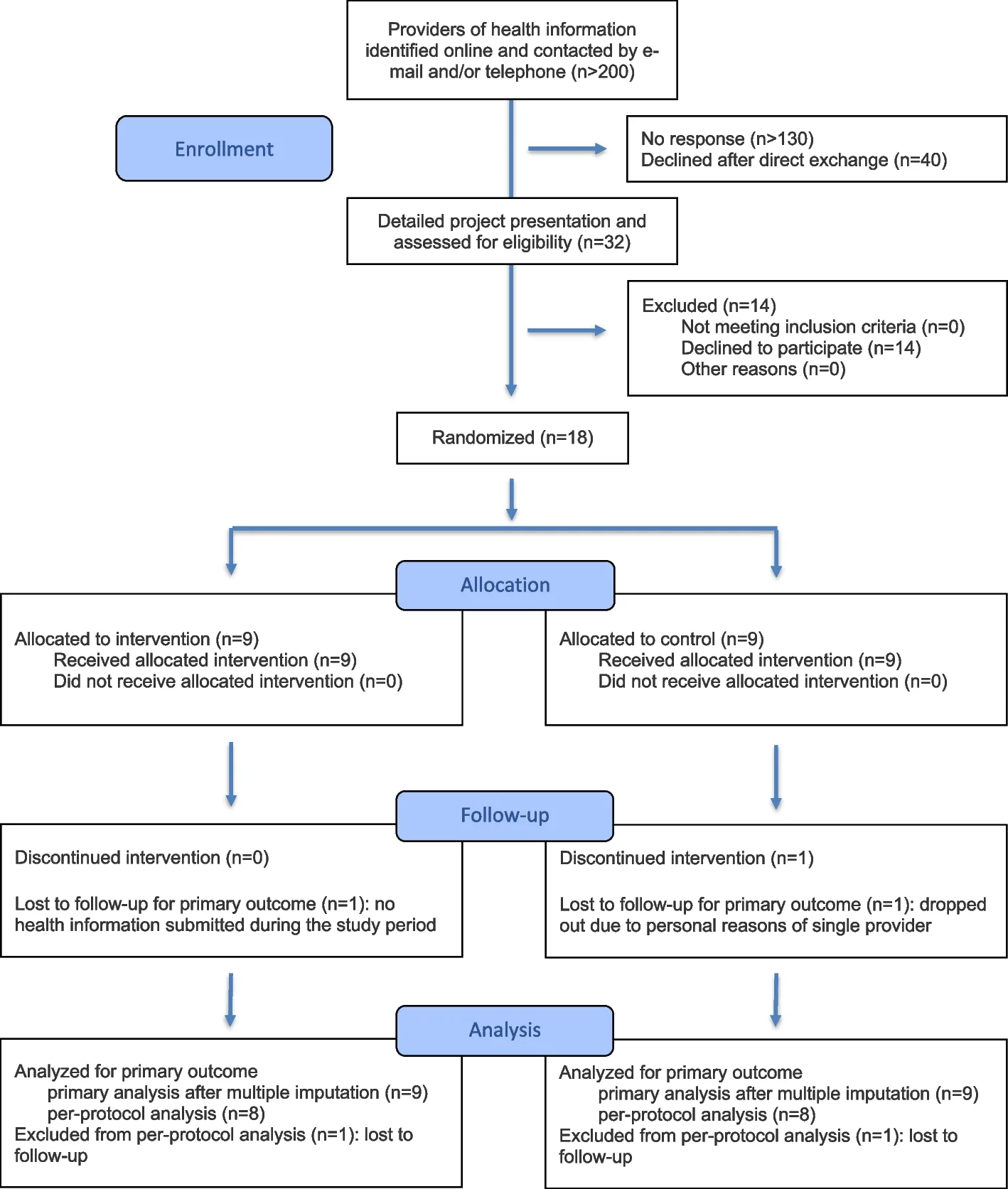
CONSORT 2025 flow diagram [33]

Between July 2019 and December 2021, > 200 providers of health information were identified. A direct exchange took place with approximately 70 providers, and more detailed project presentations were made at 32 institutions (see Fig. 1). Eighteen providers (nine IG and nine CG) were included and randomized. Fifty-four individual participants consented to participate, but only 50 (25 IG and 25 CG) completed the baseline survey. In the CG, one provider with only one participant dropped out for personal reasons. In the IG, one provider was unable to finalize health information during the study period.

Fifteen providers from Germany and three from Austria were included in this study. The amount of newly created or updated health information per year and provider varied between a low single-digit range and several hundreds. Four providers (two IG and two CG) did not produce health information on a regular basis, or at least not in this constellation but as part of projects or special assignments. Very different information was produced in terms of scope, objectives, and format (see Table 2 for the objectives). The included providers covered a wide range of topics, from general health and lifestyle information to specific, disease-related issues.

**Table 2:** Objectives of the health information of participating providers

Summary from the interviews:
- Providing evidence-based information (explicitly stated or criteria, such as independent, comprehensive, neutral, understandable, assured, and target group-orientated)
- Enhancing decision-making processes (also informed and shared decision-making)
- Improving doctor–patient communication
- Improving (critical) health literacy
- Providing knowledge about health and illness so that users can better assess symptoms or better manage their illness; raise awareness and sensitize people to an illness
- Encourage people without raising false expectations
- Passing on good experiences from self-help and turning members into experts on their illness
- Giving tips for a healthier life
- Create appealing information, attract attention, and arouse interest (“keeping readers interested”)

The size and organization of the providers’ institutions varied (from a single person to approximately 65 members of an editorial team). A maximum of 10 people participated in the project. The participants reported that the competencies for the development of health information were acquired through “learning by doing,” internships, or further education and training (also on EBM, health literacy, plain language). Health information from other providers was also critically read, and quality criteria, such as the “Good practice guidelines for health information,” were used [13]. The participants described personal interests; dialogue with colleagues, experts, and patients; and cooperation with media agencies and graphic designers as helpful. Further characteristics of the providers and participants are presented in Tables 3 and 4. The baseline values of the CHC test are presented in Table 5.

**Table 3:** Characteristics: providers of health information

**Characteristics**	**Intervention group** (*n *= 9)	**Control group** (*n* = 9)
**Sectors/institutions of providers**, *n*		
Self-help	4	0
Patient counseling	1	1
Health portals	1	4
Professional societies	1	1
Public institutions	1	2
Other	1	1
**External award of contracts**, *n*		
Yes (e.g., IT, graphic design or medical experts)	7	6
No	1	3
No indication	1	0
**Focus on specific user groups or topics**, *n*		
Yes	7	7
No	2	2
***Good practice guidelines for health information*** [13], *n*		
Unknown	4	5
Acknowledged	1	0
Basis for the methodological approach	4	4
***Guideline Evidence-based Health Information*** [14], *n*		
Unknown	6	4
Acknowledged	2	3
Basis for the methodological approach	1	2
**Selection of topics (multiple answers possible)**, *n*		
Involvement of the users	4	5
Search queries/trends/commercially driven	0	1
Current topics/news	2	2
Assignment	2	2
Own expertise and internal coordination	5	5
**Systematic literature searches**, *n*		
No	4	7
Partly (data syntheses, existing information)	4	1
Yes (medical databases)	1	1
**Involvement of the users**,* n*		
No involvement	0	0
Needs assessment		
Systematic	2	3
Unsystematic/topic identification	5	5
Feedback		
Systematic in the development process (draft)	2	3
Final information	4	4
**Funding **, *n*		
Public funds	5	4
Commercial (e.g., via advertising)	0	3
Other/mixed	4	2
**Management of conflict of interests**, *n*		
No	4	5
Disclosure	3	1
Management strategy	0	1
Not specified	2	2
Individual declaration of financial interests that could be categorized as a potential conflict, *n* (*n *individual participants)	5 (6)	6 (7)

**Table 4:** Characteristics: individual participants

**Characteristics**	**Intervention group** (*n* = 25)	**Control group** (*n *= 25)
**Sex**, *n* (%)		
Female	20 (80)	17 (68)
Male	5 (20)	8 (32)
No allocation	0	0
**Age in years**, mean (SD); range	39.5 (11.9); 21–69	32.3 (8.5); 21–54
**English language skills**, self-assessed, *n* (%)		
Level A1	5 (20)	1 (4)
Level A2	4 (16)	0
Level B1	3 (12)	5 (20)
Level B2	6 (24)	9 (36)
Level C1	4 (16)	4 (16)
Level C2	3 (12)	6 (24)
**Highest educational qualification**, *n *(%)		
Lower secondary school certificate	0	0
Secondary school certificate	3 (12)	1 (4)
Higher school certificate	2 (8)	3 (12)
University or University of Applied Sciences degree	20 (80)	21 (84)
**Knowledge of EBM**, self-assessed, *n* (%)		
Low	4 (16)	2 (8)
Moderate	9 (36)	11 (44)
Good	11 (44)	7 (28)
Very good	1 (4)	5 (20)
**Development of health information**, duration of activity in years, mean (SD); range	6.4 (5.7); 0–20	7.4 (6.8); 0–23
Use of sources for the development of health information*, *n* (%)		
Medical databases	14 (56)	18 (72)
Specialist journals	13 (52)	24 (96)
Guidelines	21 (84)	25 (100)
Experts	15 (60)	19 (76)
Other^§^	10 (40)	9 (36)

^*^Multiple answers possible; participants from one institution sometimes named different sources

^§^Other sources: exchanges with users and/or multipliers, own surveys, health information from other providers, specialist literature/books, internet, forums and blogs, legal texts, professional associations

### Quality of health information

#### Baseline

All 18 healthcare providers submitted their health information at baseline. In some cases, several health problems were addressed, e.g., diagnostics and therapy. Two diagnostic, 14 therapeutic, and two pieces of information that could not be clearly assigned were analyzed. On average, the mean values of the MAPPinfo checklist [37] were in the IG 27.8% (SD = 10.1; range, 10.0–45.0) and in the CG of 25.4% (SD = 9.8; range, 12.5–40.0). 

#### Primary outcome

Eight providers in both the IG and CG submitted their health information between December 2019 and November 2022. In the IG, one provider did not complete the information during the study period and, despite several reminders, only submitted preparatory documents. In the CG, there was one dropout, and the health information of two providers did not address a specific health problem, despite the criteria provided at the time of inclusion; therefore, not all MAPPinfo items could be used. The corresponding items were scored as 0 = “not fulfilled.” Of the remaining health information materials, 12 with a therapeutic problem, and two with a preventive problem were included in the evaluation. Scores on the provider level are displayed in Table 5.

**Table 5:** MAPPinfo scores on provider level

Intervention group				Control group			
**Provider ID**	**Individual participants [completed training], *n***	**MAPPinfo score, %**		**Provider ID**	**Individual participants [completed training], *n***	**MAPPinfo score, %**	
		**Baseline**	**Follow-up**			**Baseline**	**Follow-up**
2	4 [4]	10.0	35.0	1	1 [0]	15.0	15.0
4	4 [2]	37.5	33.3	3	1 [1]	12.5	12.5^b^
6	1 [1]	32.5	32.5	5	5 [3]	27.5	30.0
7	1 [1]	25.0	32.5	8	2 [1]	22.5	22.5
10	2 [2]	45.0	45.0	9	1 [0]	20.0	-
13	2 [2]	20.0	-	11	2 [1]	36.4	35.0^b^
14	2 [1]	25.0^a^	17.5	12	1 [1]	40.0	25.0
17	3 [3]	30.0	30.0	15	6 [4]	34.8^c^	26.1^c^
18	6 [5]	25.0	22.5	16	10 [0]	20.0	17.4^c^
Median (range)	2 (1–6) [2 (1–5)]	25.0 (10.0–45.0)	32.5 (17.5–45.0)	Median (range)	2 (1–10) [1 (0–4)]	22.5 (12.5–40.0)	23.75 (12.5–35.0)
Means ± SD		27.8 ± 10.1	31.0 ± 8.3	Means ± SD		25.4 ± 9.8	22.9 ± 7.7

Topics of health information: ^a^diagnostic problem, ^b^preventive problem, ^c^no specific health problem, all others addressed a therapeutic problem

In the primary analysis (*n* = 18 after multiple imputation), the mean values of the MAPPinfo checklist [37] were approximately 30.2% (95% CI, 24.6 to 35.9) for the IG and at 22.7% (95% CI, 17.4 to 27.9) for the CG. The difference between groups was approximately 7.6% (95% CI, −0.2 to 15.3; *p* = 0.055). By multiple imputation, 100 values were imputed in two missing values of two providers, one from IG and one from CG. Mean ± SD of 50 imputed values in each group was 23.8 ± 6.7, range 12.5–35.0 in IG and 20.5 ± 7.6, range 12.5–35.0 in CG.

**Fig. 2:**
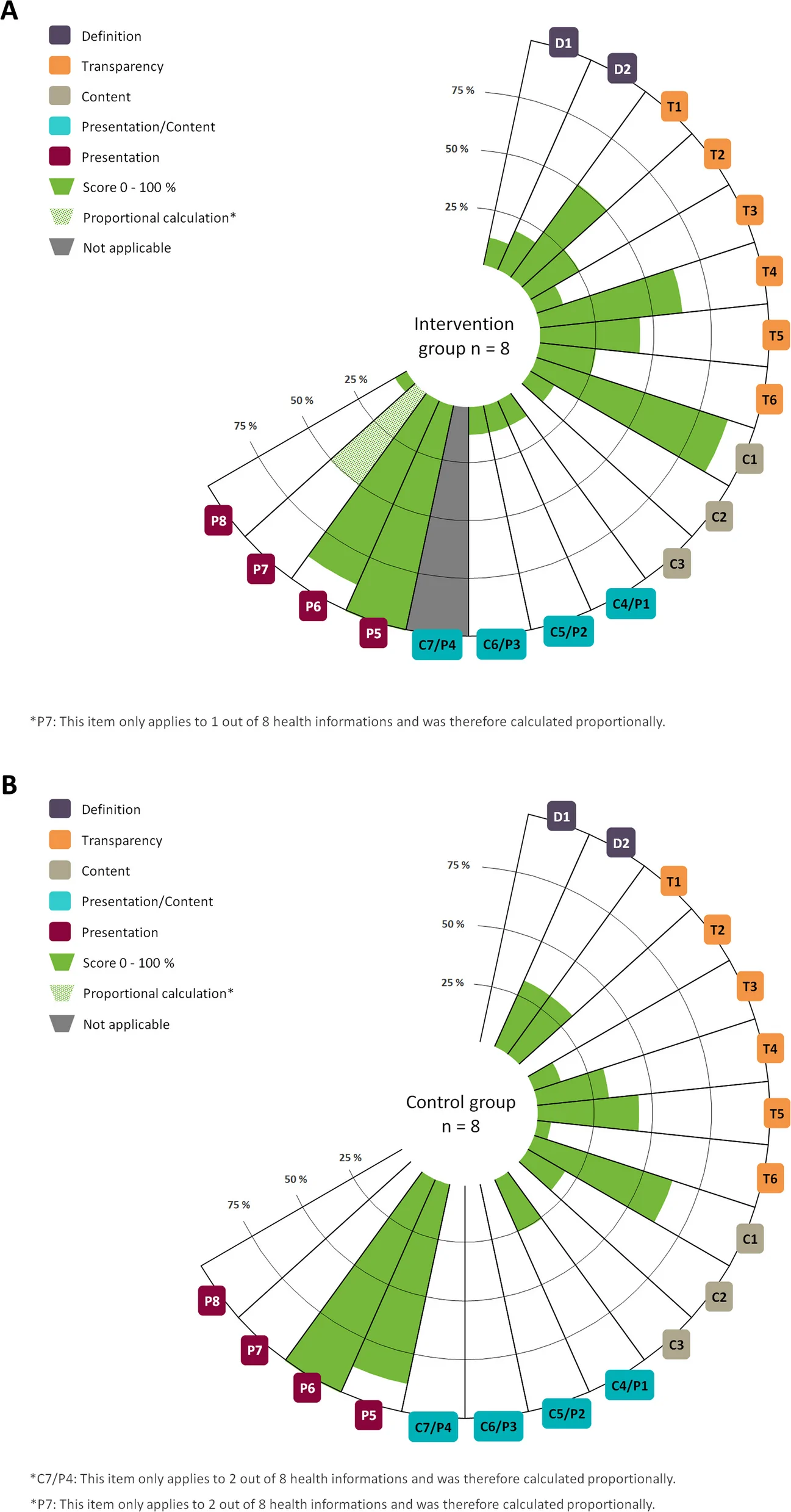
Visualization of information quality created using the MAPPinfo Evaluation Toolkit [40]. **A** Results of the intervention group. **B** Results of the control group

In the complete-case analysis, the information of the IG demonstrated a mean value of the MAPPinfo checklist of 31.0% (95% CI, 24.1 to 37.9) and the information of the CG of 22.9% (95% CI, 16.5 to 29.4). The difference between groups was approximately 8.1% (95% CI, −0.4 to 16.7; *p* = 0.0614 of the *t*-test assuming equal variances in both groups, *F*-test: *p* = 0.8519). The results of the IG and CG ratings are shown graphically in Fig. 2. ANCOVA estimated a similar group effect of 6.8% (95% CI, −0.2 to 13.9; *p* = 0.0580) as the primary analysis.

The distribution of MAPPinfo in both groups was investigated graphically using histograms; however, because of the small sample sizes, conclusions regarding normality were limited (graphics not shown). A nonparametric exact Wilcoxon test comparing both groups was performed for a sensitivity analysis, and the results were not significant (*p* = 0.0670).

#### Secondary outcomes

One out of eight providers in the IG provided information about possible benefits in an appropriate manner (C5/P2) and 0/8 in the CG. Similarly, only one out of eight providers in the IG provided information about possible harm in an appropriate manner (C6/P3) and none of the eight providers in the CG. No diagnostic problems were included in the assessment; therefore, C7/P4 did not apply to any of the information. All of the eight providers in the IG and seven in the CG used neutral language (P5).

The target group was involved in different developmental processes. In the IG, three providers systematically recorded the needs of the target group (including surveys of members of self-help groups), and four considered requests from users for the selection of topics without systematically recording specific needs. In the CG, three providers systematically recorded needs and five others considered requests unsystematically.

### Process evaluation

#### Recruitment

Despite intensive efforts, the target sample size of 26 providers could not be achieved. Most of the over 200 health information providers in Germany, Austria, and Switzerland were contacted several times via email and/or telephone. When a provider was contacted by telephone, they were asked about the person responsible for producing the health information. Direct exchanges (via e-mail or telephone) took place at approximately 70 institutions. In 32 cases, a more detailed project presentation was conducted with the persons responsible. Three took the initiative to participate in the project.

Institutions were not reached because the e-mails were unanswered, nobody could be reached by phone, or the contact details were dated. Some refused to participate after the first contact, no individual contact details of the responsible persons were provided, or it was announced that feedback was not received. Various reasons for non-participation were identified: lack of resources (e.g., to enable employees to participate in the training), lack of capacity in voluntary or part-time employment, lack of time (e.g., currently other extensive tasks, including the development of information on coronavirus disease 2019, restructuring, or participation in other projects), lack of interest (e.g., subjectively no need for training, commercial orientation that contradicts EBHI), and organizational aspects (e.g., those responsible are only involved in the provision, but not the content of the information).

Overall, the providers who participated in the project were those who actively responded directly to the invitation by e-mail or who learned about the project through other channels and made contact on their own initiative. Thus, an existing interest in the EBHI topic and a high level of motivation appear to be prerequisites for involvement in the project.

#### Training: delivery, participation, and feedback

Between August 2020 and February 2023, eight training courses were held with 17 providers (nine IG and eight CG). In total, 46 of 54 individual participants were registered on the ILIAS learning platform (not registered: six from the CG and two from the IG). Some registered participants either did not participate in the training continuously or did not complete it to the end. In the IG, 21 participants completed the training, and in the CG, 11 participants. The main reason for not completing the training was the lack of time.

A maximum of three providers participated in the training course together, and the IG and CG were also mixed. Providers of the CG participated only after they had completed data collection. Consent for joint participation in the training was verbally obtained in advance. Confidentiality was emphasized at the beginning of each training course. The exchange between the groups was generally rated as very beneficial.

Before starting, we asked the participants about their motivation to participate in the training. The answers were as follows:

- Curiosity and interest in the topic
- Acquisition of knowledge and skills and professional and/or personal development
- Desire to produce better (evidence-based) information and to be able to work scientifically
- Recognized the need for improvements in the editorial team, motivation from colleagues, and assignments that represent the institution
- Desire to reflect on one’s own work, to see where one stands and to receive new food for thought, qualified feedback, and scientific support
- Networking and exchange with other developers
- Participate in a study or support the IMLEGI project

Overall, the acceptance of the training was high. The content was rated as interesting, relevant, and easy to understand. However, the implementation of individual day-to-day work was critically discussed.

It sometimes took a little practice to navigate the ILIAS learning platform, and there were a few questions about the procedures; however, the work assignments were generally completed well. There was a request for clearer communication of the deadlines and the respective workload of the assignments, although an overview of the training program was sent out at the beginning, and the timeline was used within ILIAS to announce the next steps. However, as the modules were activated sequentially, it was not possible to provide a complete overview of the training.

Time resources and individual prior knowledge played a role in the processing of the training content. Overall, most groups required more than the planned 6–7 weeks to complete the training (14 weeks on average, max. 30 weeks). In some cases, it was difficult to find a common date for the virtual presence. With the agreement of all participants, the sessions were recorded and made available within the course.

#### Implementation of the training content: barriers and supportive aspects

Interviews were conducted with eight providers from the IG. One provider who was unable to submit information at the end of the study was also unavailable for an interview.

The results of the interviews are presented as follows:

I. Comparison of the development process with the process presented in the training

*People involved in the development process:* Between one and four people worked on each information development. Individual groups involved other experts via internal or external review processes or followed established consultation processes at their institutions. Proofreaders or designers also supported some providers.

*Identification and quality assessment of existing information materials on the topic:* This process step was conducted only once. This involved a comprehensive exploration via free Internet searches and the inclusion of a database search. However, this analysis was not reported.

*Exploration of the preferences of the target group:* The preferences of the target groups were frequently assessed. Different methods were used by the providers, such as literature searches, surveys, query analyses, and the number of hits on information portals, all of which were suitable for exploring preferences.

*Systematic identification of the evidence base*: The identification of the evidence base included determining the research questions, defining the inclusion/exclusion criteria and search strategy, selecting the databases, and screening the hits. This complex task was handled very differently both in terms of the completeness and the quality of the individual work steps. The implementation ranged from expert-based preparation to the use of existing evidence synthesis and systematic research. In some cases, the methods were not described transparently, or the process was outsourced.

*Appropriate quality assessment of studies:* Quality assessment revealed a broad spectrum of studies. Some providers used established checklists for critical appraisal and the four-eye principle. In some cases, it was not specified which instruments were used; this aspect was not reported, or it was explicitly reported that this aspect was not addressed.

*Systematic data extraction:* Data extraction was hardly reported. If this was done, the procedure was outlined with little transparency or could be described as unsystematic.

*Piloting of the health information in the target group:* Most groups obtained feedback from the target group. This was performed in different ways. They conducted interviews and unsystematically collected feedback using a website. Whether and to what extent the results were utilized was usually not clearly described. The readability indices were also calculated.

*Reflection on the procedure:* In their reflections on the processes, providers allowed insight into the extent to which the methodological approach taught could be implemented. In particular, the reflection focused on dealing with the expectations of the target group and institutional requirements and how they were handled. Individual groups were able to implement the methodological approach as planned and discussed the further development of presentation formats for health information. For others, compromise solutions were necessary, which had a strong impact on the information quality.

II. Content analysis

Eight categories with 23 subcategories were identified by content analyses. 

The eight main categories are as follows:

- Implementation of data syntheses and criteria for EBHI (six subcategories)
- Involvement of experts (two subcategories)
- Training and continuous support in the development process (three subcategories)
- Structural and institutional requirements (four subcategories)
- Conflicts and interests in the development process (two subcategories)
- Self-appraisal of the information developed in the project (two subcategories)
- Feedback on the training (two subcategories)
- Resources (two subcategories)

The following summary focuses on the identified barriers. A detailed description of the categories and subcategories with anchor examples can be found in Additional file 1.

There are barriers and facilitating factors for the implementation of the criteria for EBHI. One provider already followed the EBM methods before participating. This provider felt confirmed in many ways by the training. The participants took it as an opportunity to discuss specific aspects in greater depth (e.g., communication of frequencies). Another group had the opportunity to utilize much of the training content, as a new information format was developed and tested in parallel with this project.

Other groups reported considerable barriers to implementation. As in the case of recruitment, a lack of resources was at the center of this. The development of EBHI, particularly systematic literature searches and data extraction, was perceived as remarkably time-consuming. These additional efforts cannot be regularly afforded with the given staffing or possibilities for voluntary work. Under the study conditions, work on health information was sometimes conducted in addition to the actual tasks. Significantly better financial resources were required, as were changes in structures and work processes. The participants reported that the individual interests of those involved in the development process influenced the design of the information (e.g., relevant content had to be deleted because of a conflict between interest holders).

Even if there was a desire to provide EBHI, far-reaching changes within the institutions did not seem feasible. In particular, the provision of EBHI is not the primary objective of an institution.

Nevertheless, the participants were able to take away important aspects from the training, which, according to them, were incorporated into their daily work (e.g., critical analyses of studies on new therapies or the creation of comprehensible, neutral texts). Thus, the training program was rated positively in retrospect. However, there is also a discrepancy. On the one hand, the training was perceived as very extensive, in-depth, and therefore difficult for persons with little experience in EBM. On the other hand, the training was insufficient to develop EBHI independently. Further support for the development process is required, e.g., to discuss any difficulties that arise.

#### Critical health literacy

Overall (IG and CG), the results of the CHC test [38] show higher post-test values after completing the training program Guide2Info than at baseline (see Table 6). In the CG, only seven participants completed the post-test.

**Table 6:** Critical health literacy

	Intervention group Mean (SD)^*^, n	Control group Mean (SD)^*^, n	Total Mean (SD)^*^, n
**Baseline**	555 (105), *n* = 25	581 (91), *n* = 24	568 (98), *n* = 49
Good/very good knowledge of EBM	591 (81), *n* = 12	602 (90), *n* = 11	596 (84), *n* = 23
Moderate/low knowledge of EBM	522 (117), *n* = 13	564 (91), *n* = 13	543 (105), *n* = 26
**Post-test** (after training)	656 (212), *n* = 20	512 (249), *n* = 7	619 (227), *n* = 27

^*^Results of the CHC test, person parameters

The comparison with the EBM self-assessment showed slightly higher person parameters for self-reported “good/very good EBM knowledge” than for moderate/low EBM knowledge, although the association was not significant. One reason for the lack of significance could be the low power owing to the small number of cases.

## Discussion

This RCT did not show a significant effect of the guideline-based development of EBHI, supported by the training program Guide2Info, compared with the process without training, neither on the primary outcome, the quality of health information, nor on the secondary outcomes that consisted of the single items of the MAPPinfo checklist.

The analyses of the secondary outcomes revealed that only one EBHI provided data on the benefits and harms. However, the overall assessment remained low owing to a lack of transparency, as a website that should include the EBHI had not been developed at that time. The planned integration into this website would likely lead to a more positive assessment. Notably, the MAPPinfo checklist was not designed to map smaller learning outcomes. It is consistently constructed from the user’s perspective; that is, it follows the binary decision of whether information enables an informed decision.

The training program was rated very positively. The participants gained important insights that could be incorporated into their work. The before–after comparison of the critical health literacy showed higher post-test values after completing the training program than at baseline. However, our process evaluation identified relevant barriers at the individual and structural levels that prevented consistent implementation of the guideline recommendations and, thus, the provision of EBHI. In particular, the lack of resources and incentives, both for participation in training and the development of EBHI, represented highly relevant barriers. Commercial interests, which are associated with the need to regularly produce content and generate a high number of clicks, prevent the systematic development of EBHI. Other development groups lack the financial resources or competencies to produce EBHI. Overall, there is a lack of external incentives or binding requirements for EBHI development.

The provision of EBHI as a prerequisite for informed decision-making has repeatedly been demanded at political and legal levels and in guidelines [6]. However, recent research has shown that the available health information lacks quality. Studies that analyzed the quality of health information addressed several quality criteria. However, the results mostly showed low-quality health information, and a systematic review by Boutemen et al. showed that the readability of most mental health information on websites and in pamphlets and books did not match the competencies of the average readers [7]. The analyses of German websites that chose autism and Asperger syndrome as topics revealed that 75% provided incomplete information and only 6% of the websites showed good quality [44]. A similar study by Melis et al., who assessed the quality of 60 English and 78 Italian websites, showed comparable results. The quality of information on the English websites was higher than that on the Italian websites. Few websites provided high-quality information on all treatment options. Additionally, all websites required a high school level or higher education [45].

These findings support the need for tools that address information developers’ competencies. In 2003, the *International Patient Decision Aid Standards* (IPDAS) Collaboration established a shared evidence-informed framework to guide the development of patient decision aids and improve their quality [12]. The update was released in 2021 [46]. Han et al. described similar factors influencing the adoption of health information standards in healthcare organizations. Among other factors, cost has been described as a negative factor that makes it difficult to switch to new standards. For example, the lack of experts who can deal with or introduce new standards leads to high costs if this expertise must be purchased or trained [47].

Various initiatives have been implemented to improve health information quality. In the USA, the National Quality Forum, a not-for-profit organization, issued a set of national standards for patient decision aids based on the IPDAS. This initiative supports the Washington State Health Authority in developing and implementing a certification process. The certifying criteria include presenting a balanced approach to healthcare options based on scientific rigor and evidence [10].

In the UK, the *National Health Service* released a standard for creating health content that replaced the *Information Standard*. This standard provides guidance for creating high-quality health content and may support organizations that produce healthcare information [11].

National initiatives may support the development of EBHI and patient decision aids. Although they will not be able to eliminate all the identified barriers, an increase in high-quality health information would be a step in the right direction.

### Strength and limitations

Our study has several strengths. The development and evaluation of the complex intervention followed the UK MRC framework, including the RCT and accompanying process evaluation, to gain insight into the explanatory factors. Additionally, we used the MAPPinfo checklist, a validated instrument, to assess the primary endpoint and quality of the health information developed by the study participants [37]. The MAPPinfo checklist operationalizes the quality criteria of the *Guideline Evidence-based Health Information*.

Our study also has some limitations. The main limitation of this study was its small sample size. The small number of cases results in low power. Therefore, there may be a group difference that cannot be detected. In addition, the small number of providers and distributions in the groups leads to limitations, particularly when applying parametric methods, such as the *t*-test or analysis of covariance. However, the nonparametric Wilcoxon test showed no significant results. Despite extensive recruitment efforts, the target number of participants could not be achieved, which considerably limited the validity of the results. A major obstacle in recruitment was the lack of provider resources. In view of the generally high workload and restrictions, the consequences of the SARS-CoV-2 pandemic have also had an impact. The necessary switch to online media also meant a lack of personal contact with the study participants. However, the greater reach achieved as a result was able to compensate for this to a small extent. In addition, the MAPPinfo checklist does not map all quality criteria, but only those that are evidence-based and a prerequisite for enabling informed decision-making. However, this is associated with the restriction that the MAPPinfo checklist can only be applied to information that informs decisions. Consequently, the study participants were instructed to develop health information materials by comparing a minimum of two options for health decisions. However, the instructions were not followed completely. As the providers were free to choose the topic of the health information, the products are not easily comparable. Furthermore, it was possible to specify the topic more precisely after randomization, meaning that a post-randomization bias cannot be ruled out.

There was no concrete indication of whether, and if so, how the deviations from protocol influenced the results.

### Implications for practice

The training program Guide2Info will continue to be offered until the end of the project. Owing to the SARS-CoV-2 pandemic, we already adapted the training program to an e-learning format hosted at the *Halle School of Health Care*. Therefore, the training is currently available. The MAPPinfo checklist is freely available (www.stiftung-gesundheitswissen.de/mappinfo) and can be used by health information developers for quality assurance purposes.

## Conclusions

As long as structural barriers are not removed, training alone will be insufficient. To ensure the long-term provision of EBHI, institutional resources, workflow changes, and access to evidence-synthesis infrastructure are required. For example, the existing medical guidelines should enable the development of EBHI and patient decision aids. In addition, access to the evidence syntheses of guideline groups enables groups, such as self-help groups, to develop information. Most of these groups lack a methodological expertise in evidence syntheses.

An evidence ecosystem, which includes automated processes, aims to incorporate evidence into decision-making processes as quickly as possible [48]. The implementation also results in lower resource consumption in the longer term.

## Supplementary Information

Additional file 1: Category system: Implementation of EBHI criteria – barriers and enabling factors.

Additional file 2: The TIDier Checklist.

Additional file 3: CONSORT 2025 checklist.

## Data Availability

The datasets generated and/or analyzed in the current study are available from the corresponding author upon reasonable request.

## Abbreviations

CG: Control group
CHC: Critical Health Competence
CI: Confidence interval
CONSORT: CONsolidated Standards Of Reporting Trials
EBHI: Evidence-based health information
EBM: Evidence-based medicine
GRADE: Grading of Recommendations, Assessment, Development and Evaluation
IG: Intervention group
IPDAS: International Patient Decision Aid Standards
MAPPinfo: Mapping Health Information Quality
MRC: Medical Research Council
RCT: Randomized controlled trial
SD: Standard deviation
TIDieR: Template for Intervention Description and Replication
