# Supplementary material for: Efficacy of a training program to support the application of the Guideline Evidence-based Health Information: a randomized controlled trial and process evaluation
Source: Trials. 2026 Oct 30;27:639. doi: 10.1186/s13063-026-10057-2 (PMC13721632; doi:10.1186/s13063-026-10057-2)
Supplement: Supplementary file 1 [file 13063_2026_10057_MOESM1_ESM.docx]

Additional file 1

**Category system: Implementation of evidence-based health information (EBHI) criteria – barriers and enabling factors**

| **Categories and subcategories** | **Description of subcategory**  ***Illustrative quotes* (pseudonym of the institution)** |
| --- | --- |
| **Implementation of data synthesis and EBHI criteria** | |
| **Reflection – methodological approach** | The participants gave critical consideration to the methodological approach to the data synthesis and development of health information. With regard to data synthesis, particular attention was paid to aspects such as the formulation of a suitable research question, the appropriate search procedure, including the definition of inclusion and exclusion criteria, and the selection and evaluation of studies. With regard to the presentation, the use of graphics or supplementary tools was considered as well as general aspects for better readability.  *“We spent a long time trying to figure out what a smart, good research question is, how to develop it.”* IN11)  *“Perhaps you could add a fact box about it or something. But I don’t think we really had any questions that would have been suitable for that, did we?”* (IN25) |
| **Uncertainties in the methodological approach** | The participants also expressed specific uncertainties regarding implementation, both in terms of the methodological steps in the data synthesis (e.g., critical appraisal, data extraction) and the implementation and design of the information (e.g., content, structure, and scope of the information, communication of frequencies).  *“Well, I think the problem is that you need a great deal of know-how about how to do it, OK? I found it particularly difficult to transpose all these graphical implementations of the frequencies, i.e. to convert the numbers into a picture.”* (IN14)  *“Anyway, we had no structures about how to divide the research up or how to put the work together, go through it, and so on. That wasn’t really clear to us.”* (IN28) |
| **Reflection – learning effects** | Apart from uncertainties, the participants also saw learning effects, especially when they had managed the implementation despite the barriers described. Groups that had already started with high methodological standards felt confirmed in their approach.  “*So, then I muddled on through. And actually, writing didn’t take all that long because I already knew exactly what I wanted to say, what the studies revealed, and what conclusions they led to.”* (IN28)  *“And a lot of what was in the training course basically confirmed what we were doing, in our opinion. And in that respect, there weren't any major anchor points or leverage points for us in terms of the design.*” (IN29) |
| **Ambition vs. feasibility** | The participants had an interest in and a personal ambition to implement the EBHI criteria. However, this was not feasible for various reasons. The participants regarded this discrepancy as a deficit.  *“Things were only included that had at least randomized controlled studies with a very good quality that had been synthesized in systematic reviews. I think the standards are relatively clear. And that is included in the evaluation, the others aren’t. And I couldn’t do that in our work. And I experienced it as a deficit.”* (IN25) |
| **Who should create EBHI?** | With a view to the limited feasibility, the participants also reflected on the prerequisites needed to implement the EBHI criteria and which persons or institutions should take over the preparation. One prerequisite was considered to be scientific expertise, which could also be incorporated through collaborations or commissions.  “[…] *we have now on our Board for example, Mr.* […] *who as from now ought to be responsible for evidence-based health information. But then he says: “No, thank you.” Scientists should do this, right? And that’s a pity because I do believe that self-help already attaches importance to this. Well, we already attach importance to providing reliable information.”* (IN14)  *“And honestly, I would think it would be very beneficial to have a research center somewhere that could be commissioned to do the preliminary research*.” (IN25) |
| **Reflection – own attitude** | Reflection was also given to the own attitude towards EBHI, particularly in the context of presenting all information objectively, even if this may be alarming.  *“And there is simply not using the terminology but using plain language, soothing language, but being honest also means answering honestly. You can die in the process, if someone asks me, I'll tell them that. I think we’ve found a happy medium, although we have to mention every risk that could lead to a life-threatening condition. But quality also means that at the end of the conversation, the patient doesn't think they're going to die.”* (IN30) |
| **Involving experts** | |
| **Trust in the expertise** | The participants sometimes involved experts, especially doctors, in the process. Trust in their professional and methodological expertise was evident throughout, which is not always clear from the descriptions.  *“Well, we are working with doctors who know how systematical reviews are created and how these syntheses function and how to carry out research properly.”* (IN25) |
| **Experts’ contribution** | Experts are involved in various ways, either as advisors or as someone who originally co-authored the text. Problems also arose here when EBHI criteria had to be implemented.  *“Naturally, one can always say: “State the source here”, or something like that, right? […] The question is just to what extent the doctor or scientist, who is working voluntarily without payment, is going to make the effort.”* (IN14) |
| **Training courses and continuous support during the creation process** | |
| **Options for support and back-up** | Participants expressed the wish resp. the need to be supported during the implementation phase at the end of the training. If they are uncertain, they would like someone to talk to who could give them tips or check the results. When asked whether continuous offers of support had been accepted, limited time resources again played a role.  *“Well, to have a back-up, of course. And if in the end it's just a case of we have the graphic, but we don't know exactly how to solve the problem so that it's not misleading or something like that. And if someone with experience would take a look at it, that would of course be good.”* (IN26)  *“The prerequisite for making use of it is that you also have the time to look so thoroughly into the topic.* […] *Of course, one would always like to have someone who one can ask if there’s a problem. But the prerequisite for this is to have sufficient time to develop such questions, I’d say.”* (IN26) |
| **Further exchanges in the group** | Exchanges and discussions in a group were found to be positive and beneficial, not only during the training course but also afterwards, if desired. It was also mentioned that face-to-face possibilities were more suitable for this.  „*Yes, but in such a small group, two, three people if you could perhaps focus it somehow according to the groups: So what kind of people do I have, what kind of topics do they have? It might be appropriate if you can say the group might keep contact with each other afterwards, that would be nice, that is what I’d like.”* (IN14) |
| **Practical exercises** | Specific instructions without too much theory and practical exercises with direct feedback were also requested. The idea of a train-the-trainer module was also mentioned.  *“It would indeed be better if you had someone who could give you more concrete [instructions], where it’s not so abstract as in the course, […] Rather a kind of support while actually doing, right? Well, say,* […] *I’ve got the figures here and someone who has already done it, takes a look to see if I’ve done it correctly.”* (IN14) |
| **Structural and institutional requirements** | |
| **Cooperation (teamwork) and responsibilities** | The organization of the cooperation (teamwork) and the respective responsibilities in the institutions played a role in the implementation. A lack of support was perceived as an obstacle.  *“Well, discussing such terminologies and finding out what we wanted? We were a small group of four people. That was something that, let me say it, cost energy. But I think a quite useful result was achieved in the end.”* (IN11)  *“And it must be ensured that it is then possible for the others to somehow carry out their own work, or whatever, or something like that.”* (IN28) |
| **Participants’ skills/competencies** | The skills/competencies of those responsible were perceived as a prerequisite for support. The lack of these could lead to considerable conflicts.  *“And the actual problem was basically that our board member, who is also a medical layperson, was of the opinion that the author's text did not provide the right values."* (IN14) |
| **Directives and objectives of the institution** | Directives regarding the scope and design of the information were perceived as barriers. It was also mentioned that institutionally prescribed objectives were perceived as being partly contrary to the EBHI criteria.  “[…] *what I have already become very, very aware of is that it depends very much on the type of health information, also of how much, well, firstly how much time I have for the preparation but also of course how much space I have.”* (IN25)  *“And I don't think it's our goal in that sense. This needs to be said. Or the goal of our employer, let me put it that way. Our main goal is to advise, isn’t it?”* (IN26) |
| **Dependencies** | Dependencies were also described, e.g., the involvement of experts or sponsors who can influence the implementation of the EBHI. With regard to financing, the concern was expressed that the implementation might fail – with corresponding consequences – even if more funding were raised for an EBHI. Besides which, acceptance in the practice is needed if the EBHI is to be implemented.  *“Because then, it is existentially threatening when such unqualified objections result in the loss of funding eligibility and reputation among donors. And the fact is that we finance most of our publications from project funds.”* (IN14)  *“Well, I can produce really super pieces of health information for patients, but if the doctor doesn’t convey and distribute it, I can happily make a small bonfire with the leaflets or XY and warm my [unclear] with them.”* (IN30) |
| **Conflicts and interests in the development process** | |
| **Potential conflicts and interests (internal and external)** | Varying interests among participants and specialists led to conflicts during the development process. Participants also expressed concerns that information could conflict with external interests and, in the worst case, lead to legal action. Participants also addressed differing expectations among users.  “*And the real difficulty was basically that our board member {…] felt that the author’s text did not convey the right message. This led to a lot of arguments, both internal and with the expert.”* (IN14)  „*This was very challenging for us, not now with the diabetes but with the others, {…] How can we formulate it so that no one can sue us? Well, I wouldn’t be able to pay for a solicitor, would I?”* (IN25) |
| **Compromises** | In some cases, conflicts led to compromises that meant information could not be implemented as planned and content tended to be deleted  *“And that was almost impossible to deal with given these two opinions. Obviously, I somehow shortened it down so that both sides said, “Yes, for goodness' sake, yes, that works for us somehow.”* (IN25) |
| **Self-assessment of the information developed in the project** | |
| **Strengths** | The participants were asked about the strengths of their information. Here it was emphasized that the information addressed significant themes that otherwise would not be sufficiently or appropriately taken up. It was also stated that the information was well-founded, patient-oriented, or easily accessible.  *“And because the theme* […] *is one of the most important ones or belongs to those that appear again and again in all of the groups.”* (IN11)  *“Well, the first strength is that there is actually information to the theme and that it is so short that it can be hat it can be displayed in the praxis and read in one go*.” (IN14)  *“That we involved the patients is also very atypical for Austria and it was an attempt and, exactly, one we can learn from”.* (IN25) |
| **Limitations** | The limitations were that the aim of creating an EBHI has not been achieved, the study situation was insufficient, no clear recommendations could be given, updating is very much needed and that the information is very text-laden.  “[…] *The weakness is the execution compared to the requirement as it was intended during the execution. In terms of content, the flyer is not bad.* […] *It is just not, well, like the standard evidence-based health information should look like, I’d say.”* (IN14)  *“I believe a limitation, if you call it that, is the fact that we don’t provide a clear answer in the information, isn’t it?”* (IN26) |
| **Feedback on the training** | |
| **Scope and demands** | The training was rated positively by the participants, but the scope and demands were also perceived as challenging. Despite the scope of the training, participants with little prior knowledge in particular felt that it was not sufficient for them to implement the EBHI criteria independently.  „*And I found it particularly sad that in the end it was difficult to implement it in the praxis. I would rather concentrate on the practical aspect. Naturally, you need the theoretical background, but sometimes it was just too detailed.”* (IN14)  „*But I found it extremely lovingly put together and also very, just these different tools and means and ways, the, yes, I found a few rather demanding but it could be managed.”* (IN25) |
| **“Benefits” of the contents of the course** | Even if the contents of the training course could not be fully implemented for the development of EBHI, they were considered important and individual contents were used in practice. The training materials were also used for this purpose after the end of the project phase.  *“So, it was definitely quite exciting for me, because I really only intended and wanted to do the journalistic part and not the research part, at least originally.* *The part with the database research was the most exciting for me, because it helped demystify it a bit or showed me more concretely how it could work.* *Right*?” (IN25)  *„I do that. I take a closer look at studies and sometimes I simply describe studies so that they…I do the members' magazine.”* (IN28) |
| **Resources** | |
| **Additional time and effort required** | The creation of EBHI is perceived as very time-consuming compared to the usual procedure, especially for inexperienced providers. The additional time required was often not available, but was partially compensated for in the project through personal commitment. With a view to long-term implementation, however, limited time resources were seen as a significant barrier.  „*Yes, well, the amount of work is indeed greater than. So, if I now produce an evidence-based health information, the amount of work is already greater.*" (IN14)  „*In the sense that we simply haven't managed to really pack this mass of available information into a patient information leaflet with two people working on it part-time. It was simply impossible for us, I have to say*.” (IN26)  *“So, it won't work in practice. It's not possible, it's not feasible. It's simply not feasible with our time budget. We all have our tasks. And we can't do that on top of everything else.”* (IN28) |
| **Additional resources** | In addition to time, the participants also believe that other resources are needed, in particular adequate funding for the development of EBHI. In this respect, opportunities were certainly seen with regard to the acquisition of suitable funds. In the absence of funds, it would not be possible, for example, to deal with extensive topics appropriately.  „*But if I had the ambition, well, I would perhaps commit to it, and say that it could be included in the project funding and would perhaps also have a good chance, because it is of course also very desirable to produce evidence-based patient information, for example, for the funding body as well."* (IN14)  *„That* [low resources] *is a limitation. And to be honest, we have now also really learned which topics we can take on and which we can't, because it was sometimes very surprising which ones were so complex*.” (IN25) |
